# Baltimore College of Dental Surgery

**Published:** 1845-12

**Authors:** 


### BALTIMORE COLLEGE OF DENTAL SURGERY.

### CLINICAL INSTRUCTION.

The faculty of this institution have made arrangements by which practical instruction may be given upon the living subject, and the students may have opportunities of performing dental operations under the eye of the Professor of practical dentistry. These *cliniques*, so invaluable to the pupil— will be continued during the winter, and probably during the summer, should students residing in the city desire it. Permit me to report a sketch of the first lecture of this kind for the American Journal of Dental Science. I may from time to time report others.

Friday, Nov. 7th, 1845.—An interesting case was presented. Professor Harris remarked that the teeth of this man afforded a striking illustration of the ravages of *caries*—the crowns of the right central and lateral incisors of the upper jaw were wholly destroyed—roots remained but partially covered by the gums, which were inflamed and spongy. The left central incisor of the same jaw was hopelessly diseased : the crown of the left lateral incisor destroyed—the root covered by diseased gum. The right superior cuspidatus affected on its anterior lateral surface but not beyond preservation. The crown of the first bicuspis of the same side was destroyed, so also that of the first molar—the roots covered by inflamed and somewhat ulcerated gum. The crown of the second bicuspis had been attacked by caries but the disease had not penetrated so far as to prevent remedy. The second molar also showed disease on its anterior and grinding surfaces, but was not beyond the reach of art. The dens sapientiæ was also diseased, but not hopelessly.

On the left side of the upper jaw, the crowns of first and second bicuspides and first molar, were destroyed ; their roots invested by ulcerated gum, exhaling a fetid odor. The second molar and dens sapientiæ in a similar condition to those on the other side.

In the lower jaw the incisors, cuspidati and bicuspides were remaining, but some were slightly injured by caries. Their inner surfaces coated with tartar (salivary calculus ) The gums were inflamed, their margins thickened and slightly crowded from the necks of the teeth. The central incisors were partially loosened from inflammation and thickening of the alveolo periosteal tissues. The crowns of the first and second molars on each side had been destroyed. The dens sapientiæ had been attacked, but might be saved.

The fluids of the mouth were unhealthy and unfit for physiological purposes. The lining membrane of the mouth was slightly inflamed.

The teeth of this subject were constitutionally liable to caries—being exceedingly susceptible to the action of corrosive agents. The teeth were originally large, chalky white near their edges and grinding surfaces, and tinged with yellow near the gums. The bony substance soft, and enamel imperfect.

The Professor observed that he would have farther opportunity to discourse at length upon the cause, nature, &c. of the disease whose ravages were so strikingly manifested in this case.

The curative indications of the complication of disease here presented, were, first, the removal of the roots of all the teeth whose crowns had been destroyed and such teeth as were hopelessly diseased; 2d, to remove the tartar from remaining teeth ; 3d, the removal of carious portions of such of those as were affected by this disease, and substitution of the loss by some incorruptible metallic substance ; 4th, the replacement of the lost organs in the upper jaw with artificial substitutes. The manner of doing this will hereafter be described, and the class have frequent opportunity of performing the several operations.

The Professor then observed, that he would not detain the class longer for the present, but would give way to Professor Bond, who wished to make some remarks.

Professor Bond then addressed the class for some time, upon the indirect and constitutional effects of diseased teeth—after which the class was dismissed. R. W. C.

